# Supplementary material for: A fucose-binding superlectin from Enterobacter cloacae with high Lewis and ABO blood group antigen specificity
Source: J Biol Chem. 2024 Dec 30;301(2):108151. doi: 10.1016/j.jbc.2024.108151 (PMC11875179; doi:10.1016/j.jbc.2024.108151)
Supplement: Supporting information [file mmc1.pdf]

Supporting Information

A fucose-binding superlectin from *Enterobacter cloacae* with high Lewis and ABO blood group antigen specificity

Ghamdan Beshr[1,2,3], Asfandyar Sikandar[1], Julia Gläser[1,2], Mario Fares[1,2,3], Roman Sommer[1,2,3], Stefanie Wagner[1,2], Jesko Köhnke[1,4*], Alexander Titz[1,2,3*]

1 Helmholtz Institute for Pharmaceutical Research Saarland (HIPS), Helmholtz Centre for Infection Research, D-66123 Saarbrücken, Germany.

2 Deutsches Zentrum für Infektionsforschung (DZIF), Standort Hannover-Braunschweig.

3 Department of Chemistry, PharmaScienceHub (PSH), Saarland University, D-66123 Saarbrücken, Germany.

4 current address: Leibniz University of Hannover

*corresponding authors

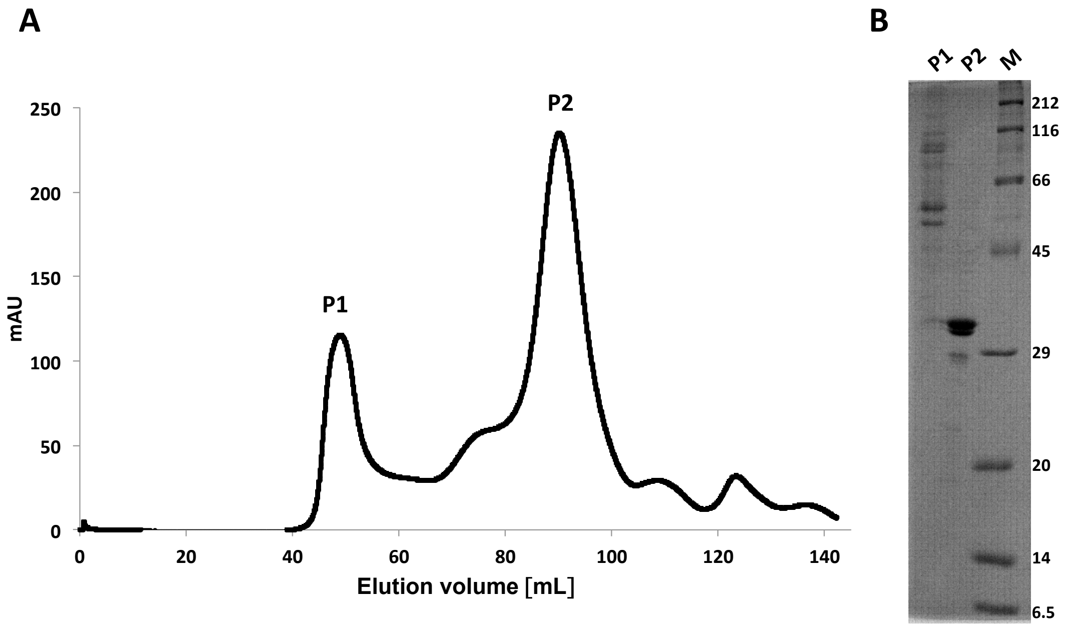

Figure S1: A. Gel filtration of culture supernatant producing EclA. B. SDS-PAGE of fraction P1 and P2 shows EclA present in P2.

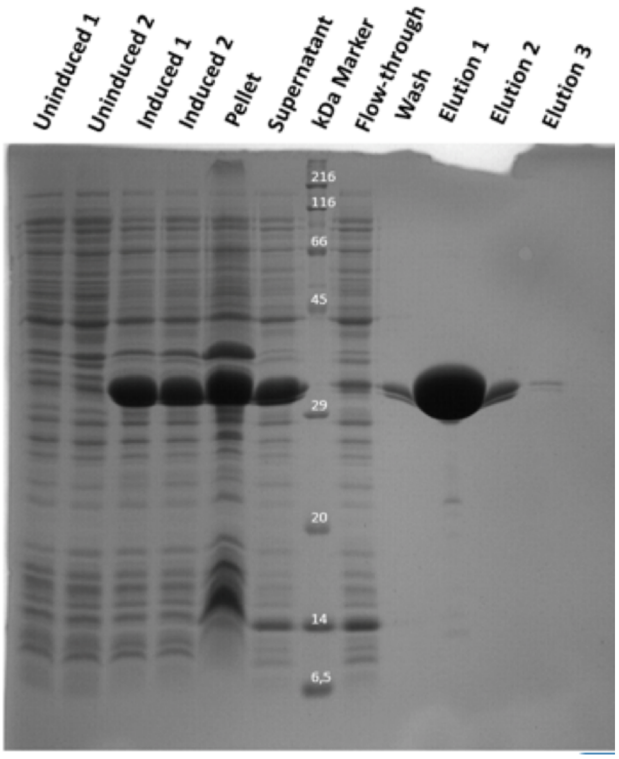

Figure S2: SDS-PAGE analysis of expression and purification of EclA on fucose-coupled sepharose resin with elution using 100 mM L-Fuc; yield: ca. 60 mg / 2 L

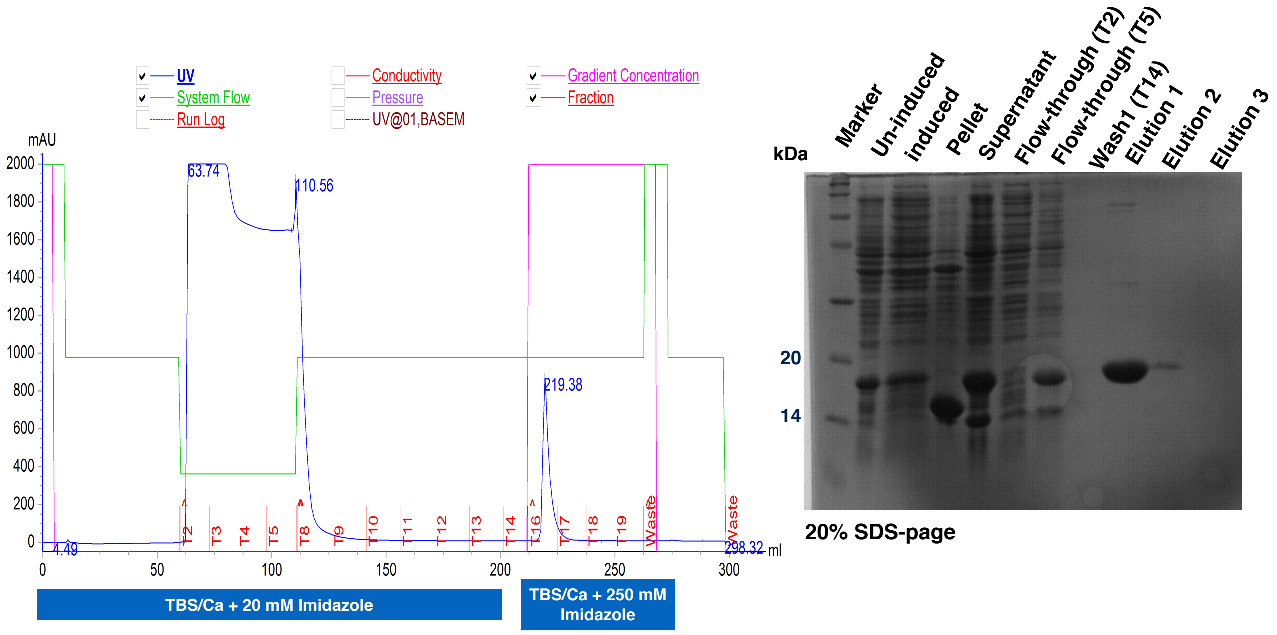

Figure S3. Expression and purification of EclA-N-tag on a HisTrap (Ni(II) sepharose) column. Left: the chromatogram of purification steps; right: 20% SDS-PAGE analysis of the expression and purification steps.

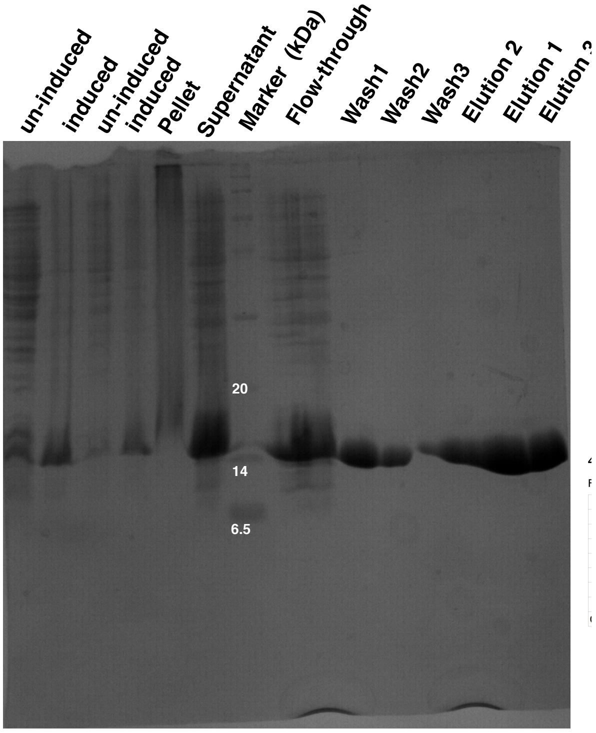

Figure S4. Expression and purification of EclA-C analyzed by 20% of SDS-PAGE.

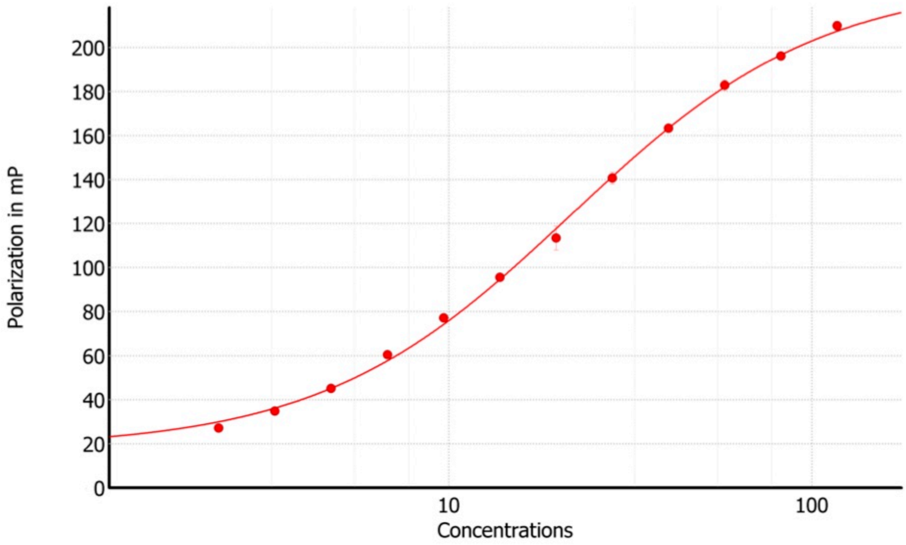

4-Parameter fit

Formula: y = Bottom + (Top - Bottom)/(1+(IP/x)^Slope)

Parameter	Value
Top	230.0
Slope	1.26032
IC50	21.732
log(IC50)	1.337
Bottom	18.0
r	0.99944
r²	0.99888
Curve Color:	

Figure S5. Direct binding of increasing concentrations of EclA-C to fluorescently labelled fucoside **11** gave a K_D of 21.7 μM. The titration was performed in 3 technical replicates.

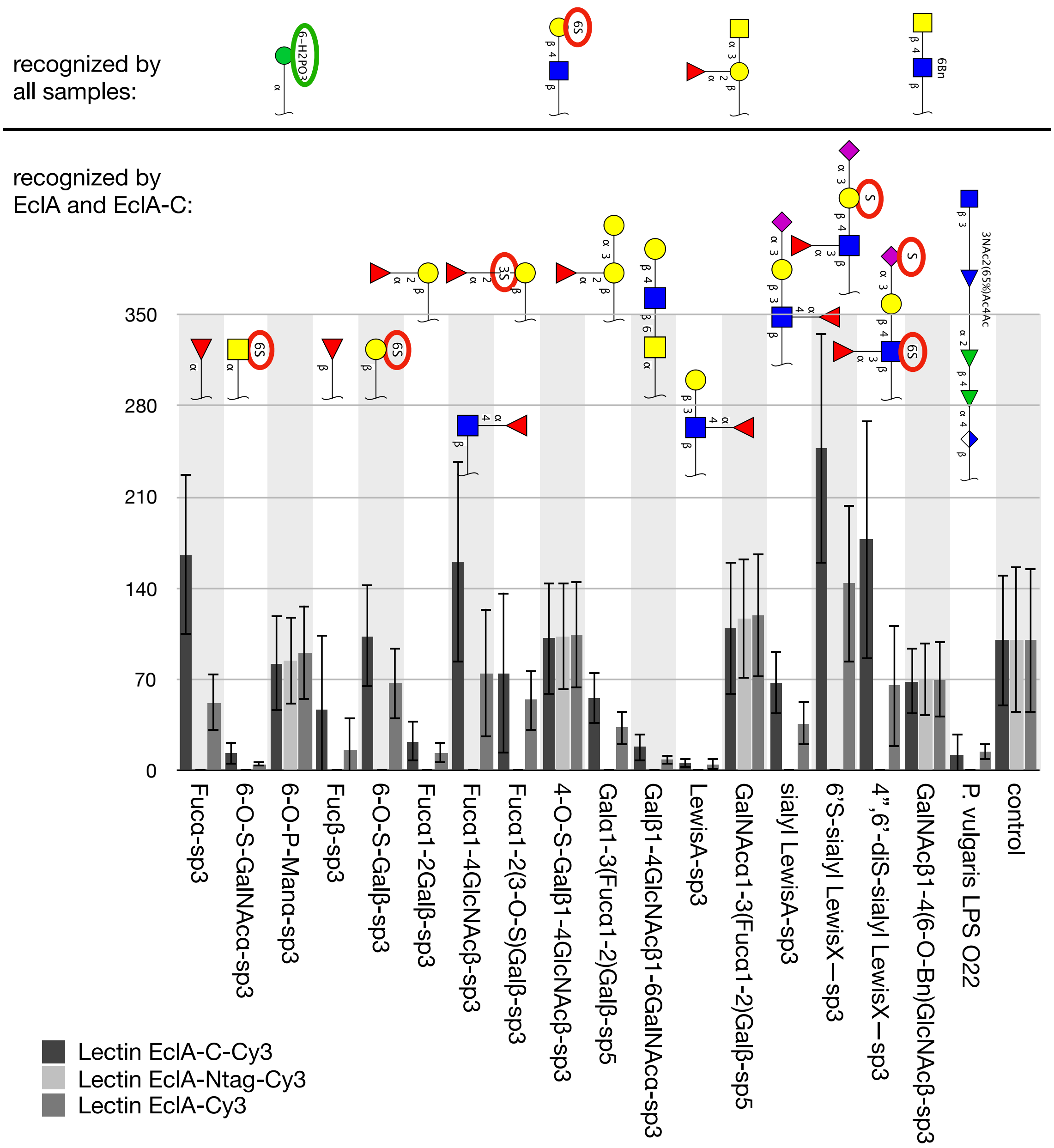

Figure S6: Analysis of Cy3-labelled EclA, EclA-N-tag and EclA-C on the Semiotik glycan array and comparative analysis of binding. The data confirmed fucose-, LewisA- and H-antigen-binding specificity for the C-terminus. In addition, a number of charged carbohydrates were bound by the constructs and four samples of which three lack fucose were equally recognized by all proteins, which contradicts CFG mammalian array and FP results. Depicted are all ligands above a fluorescence intensity threshold of 1000. y-axis is fluorescence intensity normalized to control. Error bars indicate standard deviation from technical replicates on the glycan array.

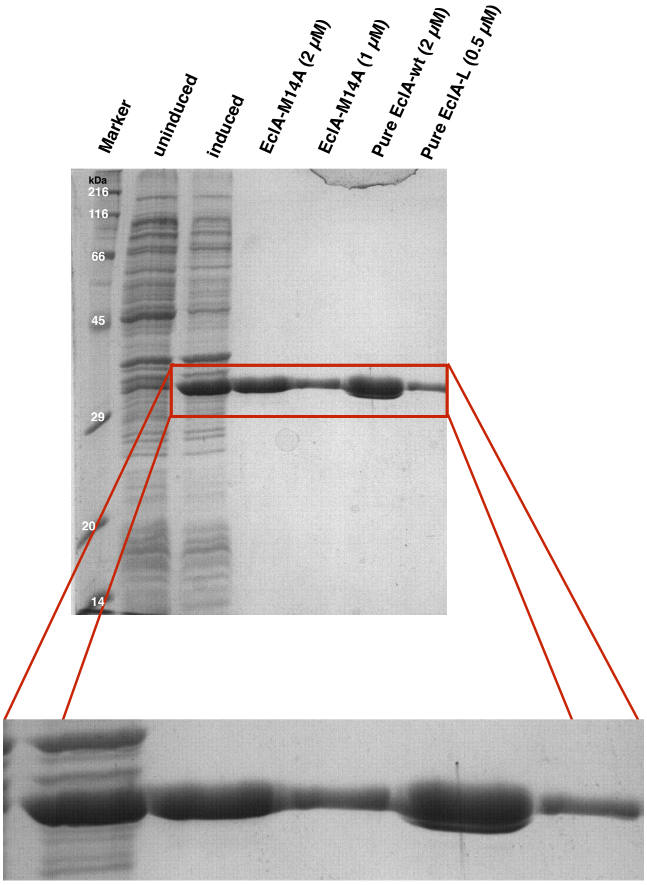

Figure S7: Expression and purification of EclA-L (EclA-M14A) shows homogenous protein species, compared to two close bands in EclA.

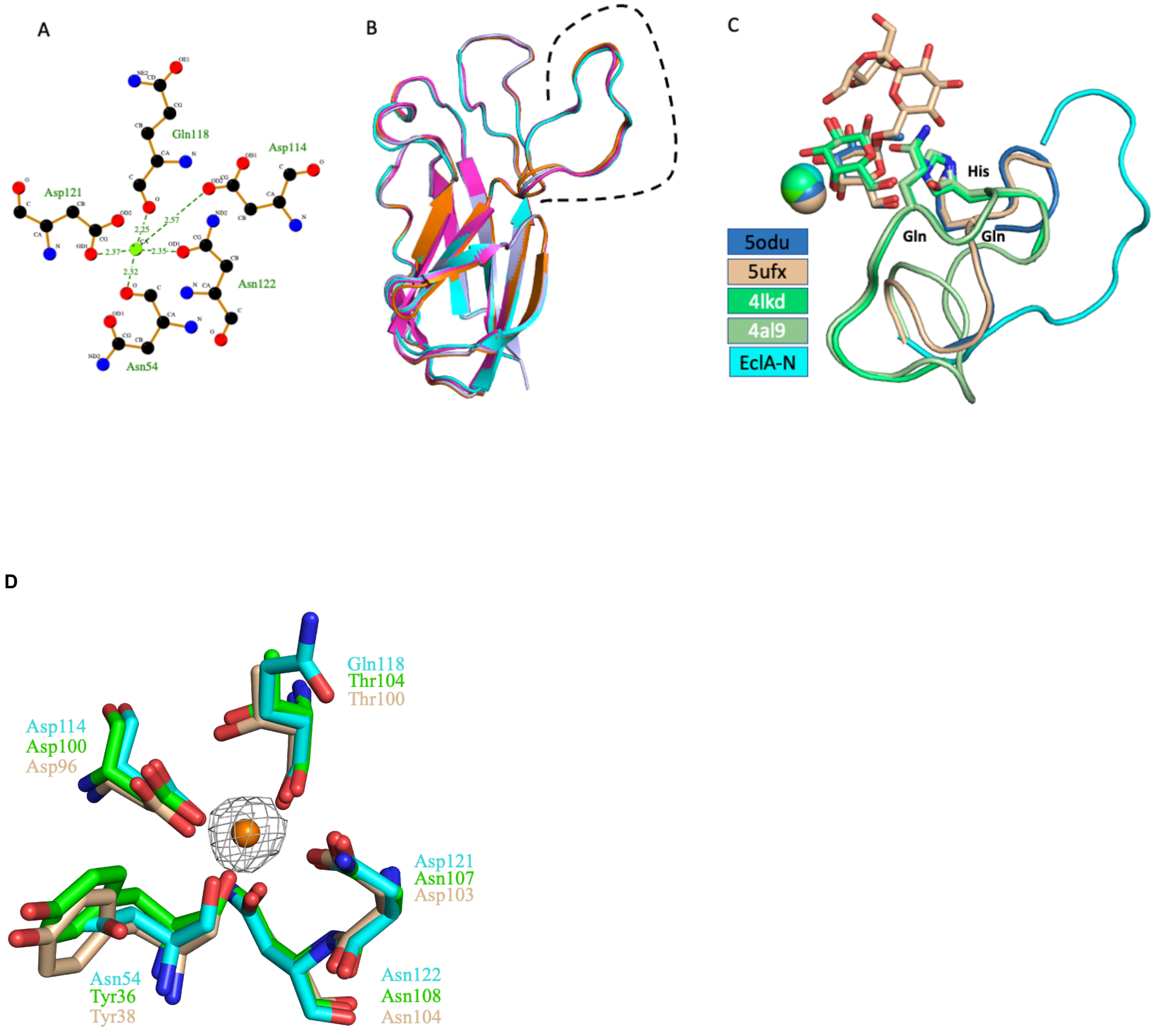

Figure S8: A. Calcium(II) coordination in EclA-N; B. Superposition of all four protomers of EclA-N in the asymmetric unit with the extended loop highlighted; C. Superposition of the binding site of EclA-N and its extended loop with galactose-containing structures of LecA (4al9, 4lkd) and PllA (5odu, 5ofx); D. Comparison of calcium ion coordination in EclA-N (cyan) with known calcium-binding lectins (PDD ID: 4al9 (LecA, green) and 5ofx (PllA, wheat)). EclA-N bound calcium ion is shown as orange sphere with electron density map (2Fo-Fc) contoured at 3α. For clarity only the residues involved in calcium ion coordination are shown

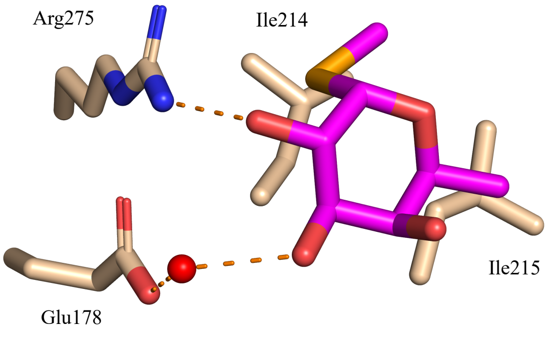

Figure S9: Schematic 2D representation of methyl α-L-selenofucoside interactions with residues of the symmetry mate. Hydrogen bonds are displayed in dotted orange lines, while all other residues exhibit hydrophobic interactions with the ligand. The water molecule is shown as a red sphere. For clarity only the interacting residues of the symmetry mate are shown as sticks.

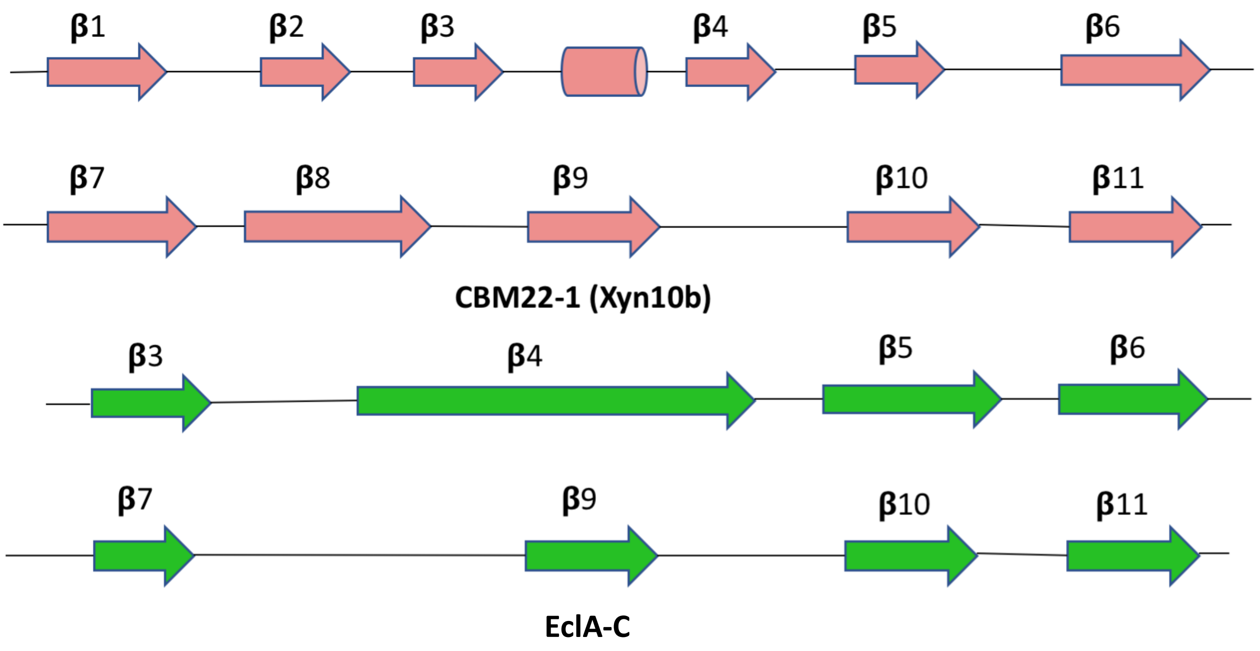

Figure S10: beta-strands in CBM22 compared to EclA-C.

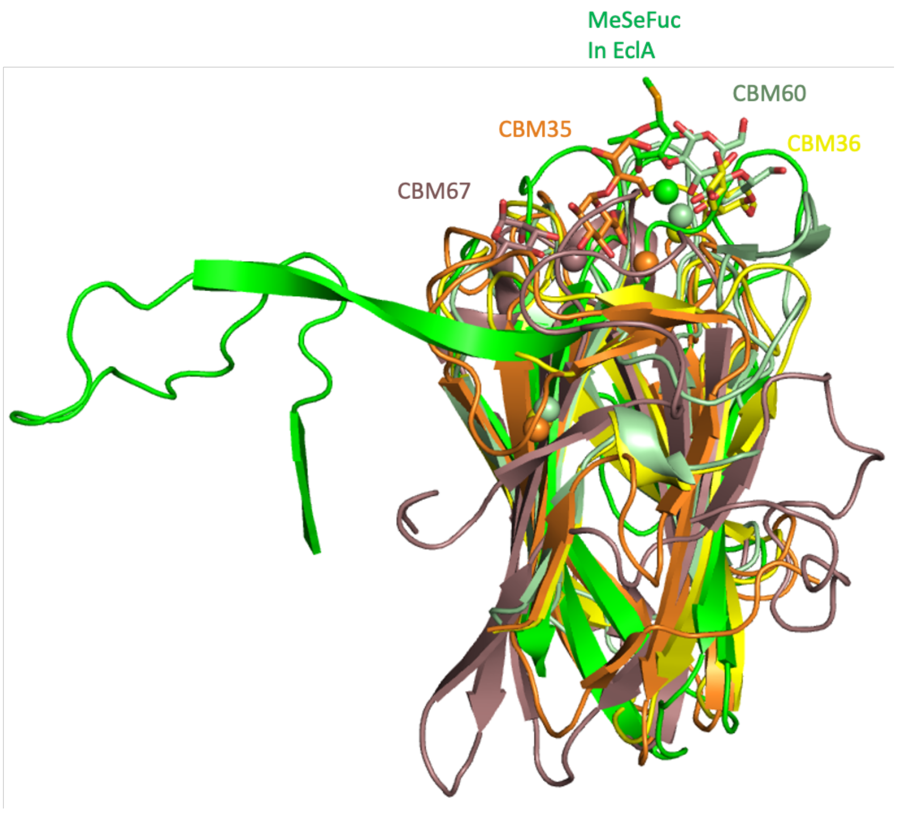

Figure S11: Superposition of the structures of EclA-C with CBM35, 36, 60 and 67 indicates calcium and ligand binding is not conserved.

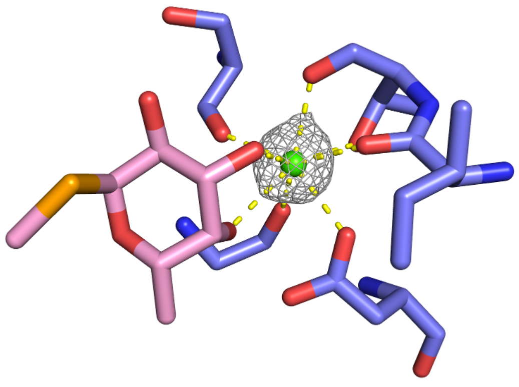

Figure S12: Metal binding site of EclA-C. Calcium ion is shown as green sphere, with electron density map (2Fo-Fc) contoured at 3α. Similar to calcium ions observed in other crystal structures (J. Am. Chem. Soc. 1996, 118, 24, 5752–5763), the calcium ion interacts with EclA-C and MeSeFuc via oxygen atoms, with a coordination number of 8. For clarity only the residues involved in calcium ion coordination are shown.

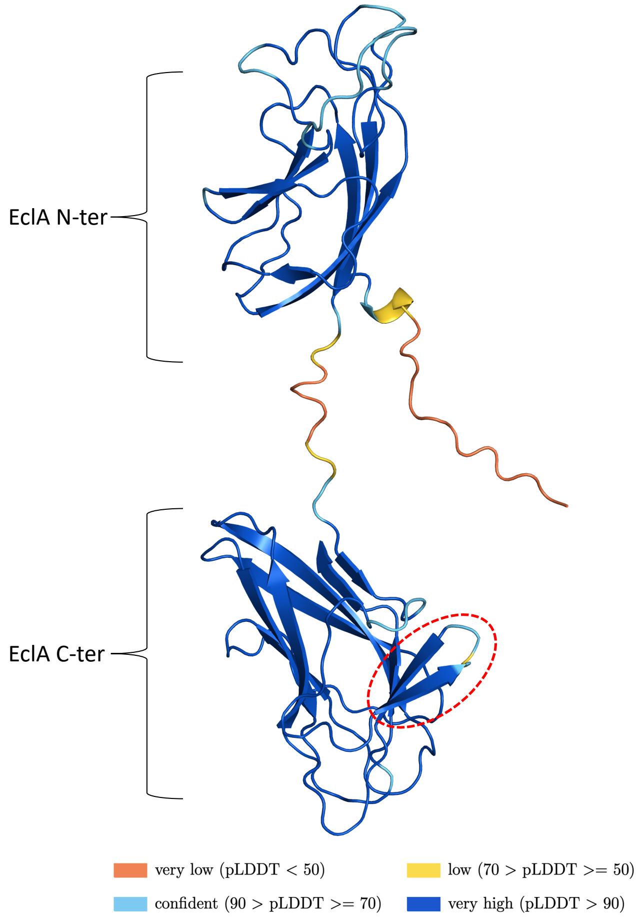

Figure S13: Structural analysis of EclA. Top AlphaFold2 prediction for EclA is shown, with residues colored according to the pLDDT score. The region involved in the β-strand swap in the EclA C-terminus crystal structure is highlighted with dotted circle (red).

Table S1. DNA Oligonucleotides used to amplify and sequence EclA-N-tag, EclA-C, EclA-L constructs by PCR.

Primer	Sequence (5'- —> -3')	Restriction enzymes	His-tag
Forward EclA-N-tag (GB36)	GGAATTCCATATGCACCATCACCATCACCATGC GAAATTCTTCGCCACGCT	NdeI	✓
Reverse EclA-N-tag (GB37)	CGGGATCCTTACGCCGCGGCGGAAATA	BamHI	
Forward EclA-C (GB39)	GGAATTCCATATGGCAGCTCCGTTTGAAGATTT AAC	NdeI	
Reverse EclA-C (GB41)	CGGGATCCTTAACCCTTTATCTTCAGTTCCTTG	BamHI	
Forward EclA-L (M14A) (GB32)	CAGACGCGAGCGAAAATTTAATCTGGTC		
Reverse EclA-L (M14A) (GB33)	TTCGCTCGCGTCTGACTCCTGTGAC		
T7 promotor	TAATACGACTCACTATATAGG		
T7 terminator	GCTAGTTATTGCTCAGCGG		

Table S2. Protein crystallography data collection and refinement statistics.

	EclA-N-tag	EclA-C
**PDB ID**	6YGQ	6YF6
**Data collection**		
Space group	P 1 2_1 1	P 2_1 2_1 2_1
Cell dimension		
a, b, c (Å)	65.79, 50.46, 73.86	38.51, 63.67, 134.01
a, b, g (°)	90.00, 109.36, 90.00	90.00, 90.00, 90.00
Wavelength (Å)	1.00	0.967
Resolution	48.87 – 1.90 (1.94 – 1.90)*	46.16 – 2.00 (2.05 – 2.00)*
Rsym or Rmerge	0.066 (0.330)	0.064 (0.321)
Rpim	0.064 (0.300)	0.018 (0.108)
CC (1/2)	0.99 (0.88)	1.00 (0.99)
I / sI	8.0 (2.2)	35.6 (8.5)
Completeness (%)	99.9 (99.7)	99.6 (96.3)
Redundancy	3.4 (3.4)	25.6 (18.4)
**Refinement**		
Resolution (Å)	40.20 – 1.90	36.57 – 2.00
No. reflection	36245 (3571)	22932 (2175)
Rwork / Rfree	0.182 / 0.225	0.193 / 0.241
No. atoms	4082	2471
Protein	3700	2200
Ligands	4	59
Solvent	378	240
Protein residues	480	280
B-factors	26.91	39.17
Protein	26.03	39.63
Ligands	35.54	29.08
Water	35.54	36.26
R. m. s deviations		
Bond length (Å)	0.003	0.003
Bond angels (°)	0.63	0.60
MolProbity score	1.07	0.83
*Values in parentheses are for highest-resolution shell		
